# The role of the Accredited Social Health Activists in effective health care delivery: evidence from a study in South Orissa

**DOI:** 10.1186/1753-6561-6-S1-P1

**Published:** 2012-01-16

**Authors:** Arima Mishra

**Affiliations:** 1 Institute of Public Health, Bangalore, India

## Introduction

In order to provide effective healthcare to the rural population, the National Rural Health Mission (NRHM) by government of India proposed introduction of female health workers village level. These workers are called Accredited Social Health Activists (ASHA) and their role is to act at an interface between the community and the government healthcare services. More specifically, she is responsible for promoting universal immunisation, referral and escort services for reproductive and child healthcare and other health care delivery programs.

In this context, this paper would discuss the role of the ASHAs in fulfilling these responsibilities and thus facilitating access to quality health care in rural areas. The paper draws on data collected as part of a larger research project on “Explaining differential immunisation coverage in India and Malawi”, a collaborative project between University of Oslo (Norway) and University of Delhi (India).

## Methods

We collected data using anthropological tools including (1) observing and following up on the ASHA's role on Immunisation days, and escorting the pregnant woman to the healthcare institutions for deliveries; (2) attending training of ASHA and their monthly meetings; and (3) conducting in-depth interviews with ten ASHAs working in different villages. We did fieldwork in Koraput district of Orissa from May 2010 to September 2010.

## Results

We found that Auxilary Nurse Midwife (ANM) of respective health sub-centre nominated all ASHAs for recruitment and hence the involvement of the community and village panchayat (elected local governance body) in selections of ASHA was very limited. Hence ASHA looked upon themselves as another cadre of state healthcare services accountable to the medical supervisor in the primary health centre rather than the Panchayat and the community. This certainly hampered the primary role of the ASHAs.

We found that different ASHAs understood their work profile differently. Those ASHAs who also worked in non-government organisations or were a member of the self-help groups set a larger work profile for themselves than others. One common distinctly visible role of all the ASHAs was 'taking care of the pregnant women' which essentially included promoting three pre-natal check-ups and institutional delivery. We observed that the cash incentives have certainly increased the number of institutional deliveries as the ASHAs make all possible efforts to take the pregnant woman to the healthcare institutions for delivery. We also observed that the immunisation coverage has also increased as the Immunisation days were conducted on a regular basis in the village.

We observed that the cash incentives (for all the work done by ASHA) however have several unintended consequences. It diluted the focus on safety associated with institutional delivery. The ASHAs along with other health workers encouraged pregnant women to deliver in the healthcare institution solely on grounds of monetary benefits. ASHAs preferred to work in several villages as they translated it as 'more villages, more cases and more money'.

Other constraints towards institutional delivery including the distance and limited healthcare facilities available in the primary health centres, and no facilities whatsoever at health sub-centres affected ASHA’s role. Community responded to the ASHAs better when they offered a range of services including curative services (e.g. medicines for common ailments like malaria, diarrhoea). ASHA coordination meetings mainly limited to submission of health records than discussing field experiences.

## Discussion

Our findings imply that there should be more involvement of community in recruiting and discussing responsibilities of the ASHAs. This will enable ASHAs to effectively act as a bridge between the community and the formal healthcare services.

Strengthening the healthcare institutions, by better equipping health sub-centres and primary health centres for safe childbirth services, can enhance ASHAs’ contribution. This is because referrals from healthcare services to long distance hospitals act as a major deterrent for institutional deliveries. Also there should be more scope for discussion and feedback among the frontline health workers and the supervisory staff.

